# SELUN: a high-speed X-ray photon counting detector for coherent imaging applications

**DOI:** 10.1107/S1600577525009026

**Published:** 2026-01-01

**Authors:** G. V. Montemurro, S. Grimm, T. Donath, P. Zambon

**Affiliations:** a DECTRIS Ltd, Taefernweg 1, 5405Baden-Daettwil, Switzerland; University College London, United Kingdom

**Keywords:** SELUN, photon counting, hybrid pixel detectors, count rates, coherent diffraction imaging

## Abstract

In this article we present SELUN, a novel X-ray photon counting hybrid pixel detector developed at DECTRIS Ltd for coherent diffraction imaging techniques at synchrotron facilities. Its notable features are a pixel size of 100 µm × 100 µm arranged in a matrix of 192 × 192 elements, the possibility of use of both silicon and high-*Z* sensors to guarantee high quantum efficiency across a wide range of incoming X-ray energies, fast front-end electronics equipped with instant retrigger technology working in non-paralyzable counting mode, and high frame rates capability up to 120 kfps thanks to two on-chip data-compression mechanisms. Optimized towards speed to cope with the enhanced brilliance of fourth-generation synchrotron sources, it shows remarkable count rate saturation values ranging from about 30 to 60 Mcts s^−1^ pixel^−1^, depending on the sensor material and value of the incoming X-ray energy, and energy-resolution figures of 664 eV r.m.s. for a silicon sensor and 1.22 keV r.m.s. for a cadmium zinc telluride (CZT) sensor.

## Abstract 2

Here, we present SELUN, a novel X-ray photon counting hybrid pixel detector developed at DECTRIS Ltd for coherent diffraction imaging techniques at synchrotron facilities.

## 1. Introduction

The advent of diffraction-limited storage rings and fourth-generation synchrotron sources is paving the way for significant advancements in terms of low beam emittance, high brilliance and high degree of coherence (Chapman, 2023 ▸). These kinds of facilities, based on the use of multi-bend achromat design (Borland *et al.*, 2014 ▸), can be found as of today at MAX IV Laboratory (Tavares *et al.*, 2014 ▸), Sirius (Liu *et al.*, 2014 ▸), ESRF–EBS (Raimondi, 2016 ▸), Advanced Light Source (Borland *et al.*, 2018 ▸) and Swiss Light Source (SLS) at PSI (Streun *et al.*, 2018 ▸), while Diamond II is undergoing upgrades in this direction. The high degree of coherence is opening the doors for revolutionizing X-ray imaging techniques, such as ptychography, Bragg coherent diffraction imaging and X-ray photon correlation spectroscopy (XPCS) (Thibault *et al.*, 2014 ▸; Förster *et al.*, 2019 ▸), but the associated high brilliance, up to a factor 100 higher with respect to third-generation synchrotron sources, is pushing the currently available detectors to the limit of their performance. In particular, the capability of handling the enormous dynamic range of the incoming beam (Leonarski *et al.*, 2023 ▸) and the capability of allowing for higher output data rates (Cipiccia *et al.*, 2024 ▸).

To overcome these limitations, we developed SELUN, a novel X-ray hybrid photon counting detector specifically designed towards high count rate and high frame rate performance. The choice of operating the detector in photon counting mode was taken in consideration of the number of advantages known to have dictated the success and widespread of such technology in the last couple of decades. Primarily, the absence of noise associated with the pixel readout operation^1^, no dark counts^2^, high overflow-free counting dynamic range, short readout time, narrow point-spread function, high quantum efficiency, high count rate capability (up to several Mcts s^−1^ pixel^−1^) with single photon accuracy, frame rate on the kilohertz range allowing for rapid correlation times and scanning speeds, and stability of operation (Donath *et al.*, 2023 ▸; Narayanan, 2024 ▸; Batey *et al.*, 2022 ▸). SELUN’s main features are a pixel size of 100 µm × 100 µm in a gapless matrix of 192 × 192 elements; the possibility of using both silicon and high-*Z* sensor materials for optimal coverage of a wide energy range; a fast front-end electronics equipped with instant retrigger technology, allowing for a specific type of non-paralyzable counting mode up to about 90 Mcts s^−1^ pixel^−1^; maximum frame rates of 120 kfps, achieved through a combination of a fast parallel readout architecture and two independent on-chip data-compression mechanisms, namely floating-point counter encoding and 2 × 2 pixel digital binning. First tests on a SELUN prototype were already reported by Cipiccia *et al.* (2024 ▸) in the context of an X-ray ptychography experiment for life science. There, it was demonstrated how its enhanced count rate and frame rate capabilities with respect to a previous-generation state-of-the-art detector (DECTRIS EIGER2) can potentially lead to a significant step forward in terms of both data quality (spatial resolution) and reach of the experiment (magnitude of the imaged sample).

In this work, we will (i) provide a basic description of the application-specific integrated circuit (ASIC) functionality and mode of operations; (ii) present the choice of the sensor materials, namely (standard) silicon and cadmium zinc telluride (CZT); and (iii) show a collection of characterization results, comprising both sensor types and delving into the spectral performance, threshold energy calibration, threshold-trimming accuracy and count rate capabilities.

## 2. Materials and methods

### 2.1. ASIC

**Figure 1:**
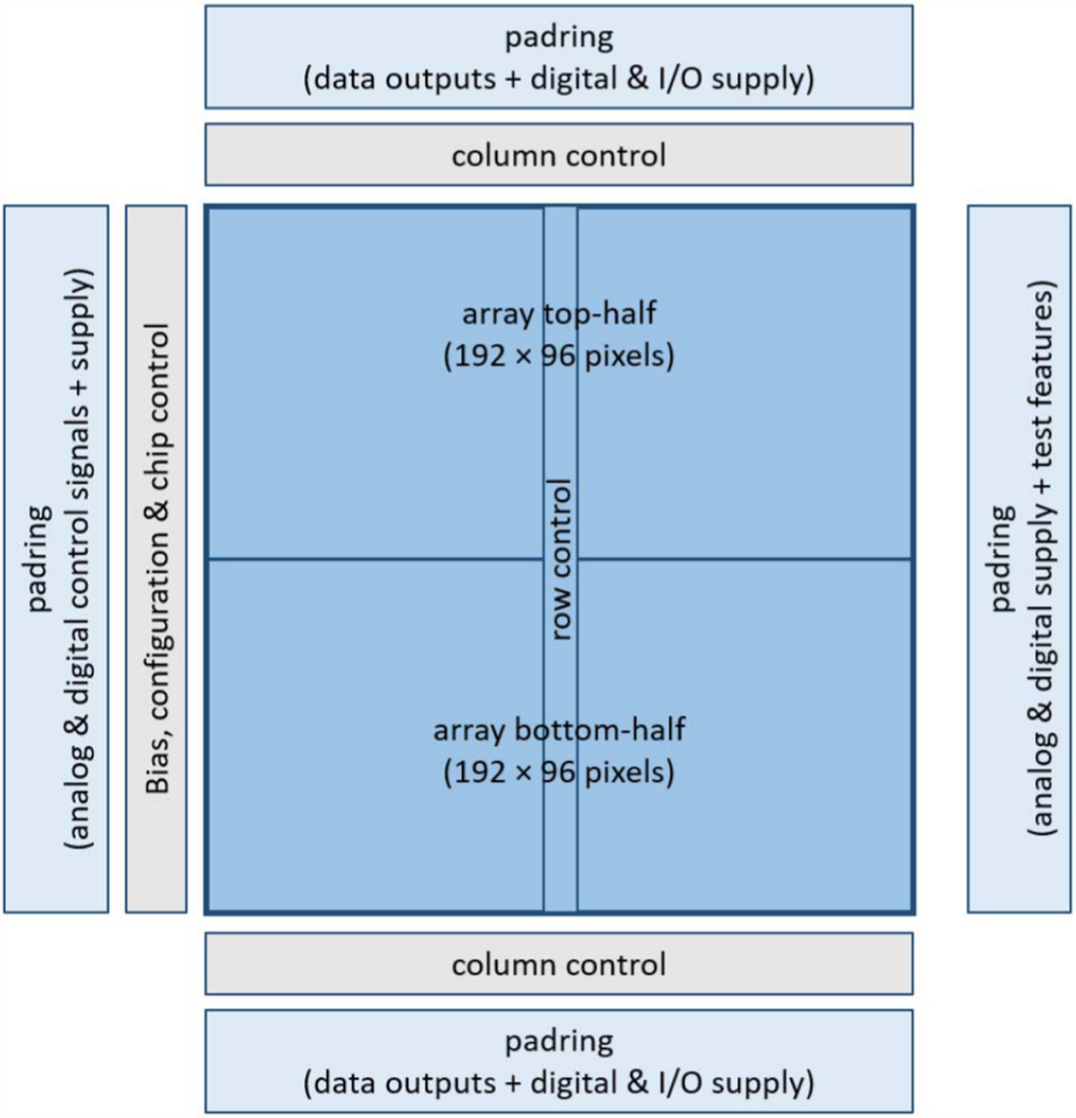
Simplified block diagram of the ASIC.

The photon counting readout ASIC is constituted by a matrix of 192 × 192 pixel elements, each with a physical size of 100 µm × 100 µm. The total chip dimension is about 2 cm × 2 cm, including ancillary electronics and wire-bond pads for power, control and data transmission, located in the device periphery. Fig. 1 ▸ shows a high-level block diagram of the ASIC, comprising three main blocks organized in a modular and hierarchical structure: the pixel array; row, column and configuration control blocks; and the I/O pad ring.

The first stage of the front-end electronics of each pixel consists of a charge-sensitive amplifier (CSA). The capability of accepting bipolar input signals allows for holes collection, as required by standard silicon sensors, and for electron collection, as required by the most commonly used high-*Z* sensors – namely gallium arsenide (GaAs), cadmium telluride (CdTe) and CZT. The feedback network includes a metal oxide semiconductor field-effect transistor (MOSFET) operated in the ohmic regime to provide a path for the sensor leakage current and for a fast signal discharge. Optimized towards speed, the CSA can output signals with a temporal amplitude of the order of 10 ns full width at half-maximum. The operating point of the feedback transistor can be tuned to match the desired stage gain. The CSA directly feeds a single-ended differential amplifier in open-loop configuration, acting as comparator for the thresholding mechanism. The global threshold value, common for the whole array, can be adjusted and equalized with a six-bit digital-to-analog converter (DAC) trimming circuitry provided to each individual pixel. The instant retrigger technology (Loeliger *et al.*, 2012 ▸) allows the system to work in a peculiar type of non-paralyzable counting mode by counting the time-over-threshold of piled-up signals in multiples of a predefined and selectable retrigger time τ_R_. The retrigger circuitry block, which can be enabled or disabled by an internal register, allows for a sensible extension of the response linearity range. With an achievable retrigger time as low as ∼10 ns, the count rate saturation occurs at about 10^8^ counts per second per pixel (cts s^−1^ pixel^−1^).

**Figure 2:**
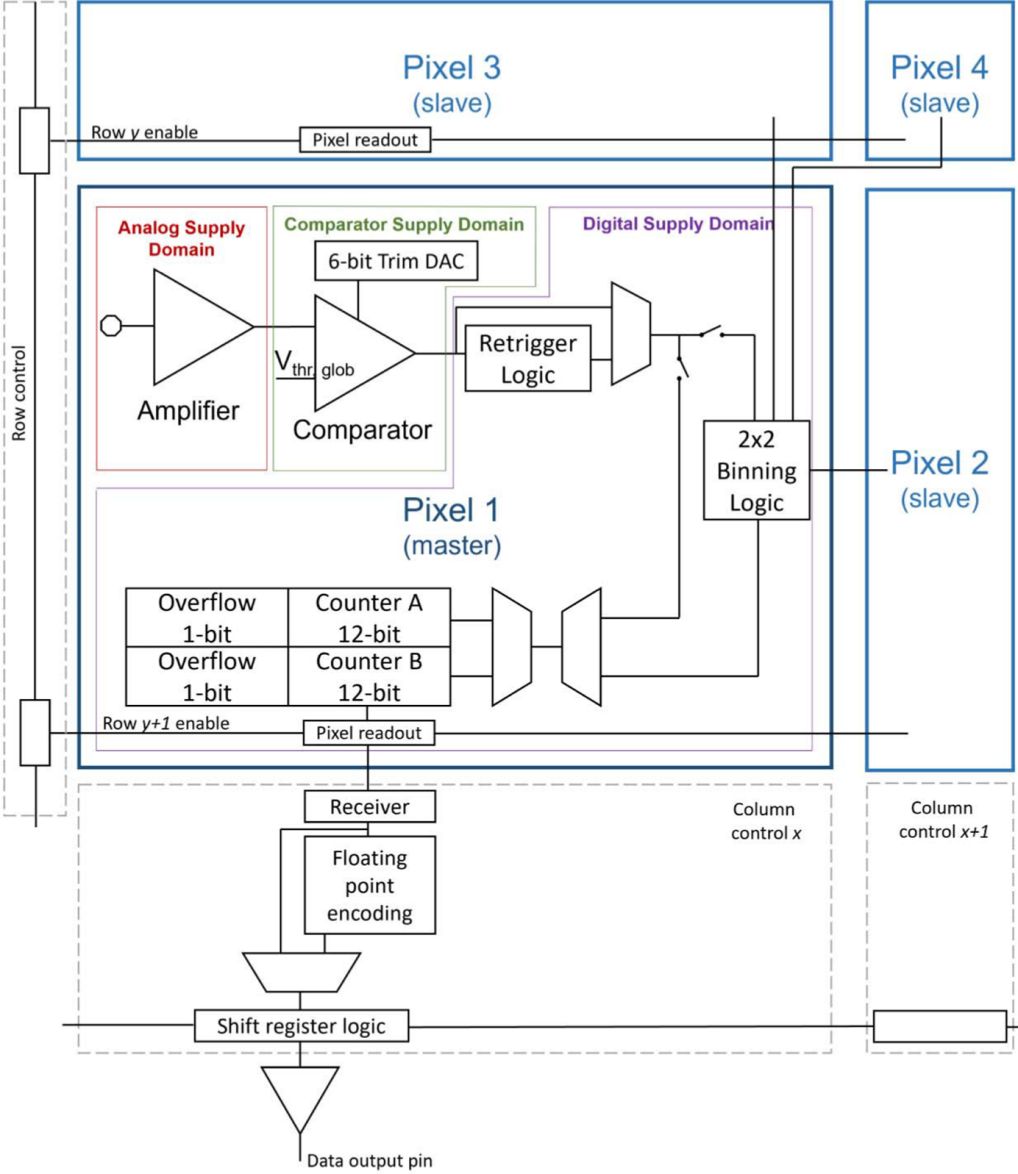
Simplified block diagram of the signal readout scheme. Pixel 1 to pixel 4 form a representative macro-pixel, with pixel 1 as the master. The architecture of pixel 2 to pixel 4 is the same as pixel 1, except that the output of the multiplexer after the retrigger logic goes either to the local counter or to the binning logic of pixel 1. The readout occurs row-by-row.

The comparator and retrigger blocks feed the pixel counter logic consisting of two 12-bit ripple counters. By writing and reading alternately the two counters^3^, a continuous readout operation is achieved. To give the possibility to achieve higher frame rates, two data-compression strategies have been implemented on-chip, at the cost of minor compression error. The first is floating-point counter encoding. It converts the 12-bit counter value from an integer to an 8-bit floating-point representation using 5 bit for the mantissa, 3 bit for the exponent and the technique of the implicit leading bit. The conversion is performed during the readout phase in the ASIC periphery with negligible time overhead. This stratagem leads to a compression ratio, and therefore a speed-up factor, of 3/2. The relative error due to the discretization introduced by the floating-point conversion is below ∼3% over the entire counting range, and in any case is not higher than the variance of the Poisson statistics of the incoming beam. A more detailed study of the discretization error is given by Zambon *et al.* (2023 ▸) in the framework of a study of the KITE ASIC, which implemented the same solution. The second data-compression strategy is 2 × 2 pixel digital binning. This in-pixel mechanism sums up the digital counts of four neighboring pixels (arranged as a squared macro-pixel) into a single 12-bit counter. The compression ratio, and the corresponding speed-up factor, is thus 4, at the cost of a reduced spatial resolution. The two mechanisms can be enabled independently and simultaneously, allowing one to reach an overall compression ratio and speed-up factor of 6. Fig. 2 ▸ shows a simplified schematic diagram of the signal readout from the front-end electronics of a representative pixel to the corresponding serial data output pin.

The static power dissipation amounts to 560 mW for the analog domain, 480 mW for the comparator domain and 7 mW for the digital domain. The dynamic power dissipation, on the other hand, can reach at most ∼4 W.

Table 1 ▸ summarizes the key technical parameters of the ASIC.

### 2.2. Sensor

There were two sensors used in the context of this work. First, a standard p-on-n silicon sensor with a thickness of 450 µm for the lower X-ray energy range. Second, a CZT sensor with a thickness of 1500 µm for the higher X-ray energy range. The choice of CZT as the high-*Z* sensor instead of the more common and mature CdTe was driven by its superior robustness against polarization phenomena induced by high charge injection levels reached at high X-ray dose rates (high flux and photon energy) (Cline *et al.*, 2024 ▸). Both sensors feature the same pixel size of the readout ASIC, *i.e.* 100 µm. Also, relatively high bias voltages were chosen to guarantee fast carrier drift times to the collecting electrodes, thus reducing charge-sharing effects due to thermal diffusion and possible ballistic deficit due the very fast shaping time of the readout electronics that would slow down the count rate performance.

### 2.3. Experimental setup

The readout ASIC and sensor assembly were glued to a carrier PCB and housed in a metal frame with a hollow pipe serpentine connected to a chiller for active water cooling at 25°C. The ASIC was read out to a series of boards connected to a Linux server for control (through Python API) and data storage via a single 10 Gb s^−1^ optical fiber cable. This bandwidth allows for a base frame rate of 20 kfps in normal mode and up to a maximum of 120 kfps with both data-compression mechanisms enabled.

The experimental characterization was carried out both in our in-house laboratory and at external facilities. Our in-house laboratory is equipped with a GE ISOVOLT TitanE 160 kVp X-ray tube with a W anode. Basic characterizations and calibration were performed by exploiting fluorescence radiation from a set of elemental targets. Count rate measurements were, on the other hand, obtained during a measurement campaign at the BAMline of the BESSY-II synchrotron of Helmholtz-Zentrum Berlin (HZB), in collaboration with the Physikalisch–Technische Bundesanstalt (PTB) Synchrotron Radiometry department (Görner *et al.*, 2001 ▸). This beamline’s salient characteristic is the possibility to deliver monochromatic X-ray energies in the range 8–60 keV of known flux, computed using a silicon photodiode calibrated against a cryogenic radiometer as primary standard (Gerlach *et al.*, 2008 ▸).

## 3. Results

### 3.1. Spectral performance

**Figure 3:**
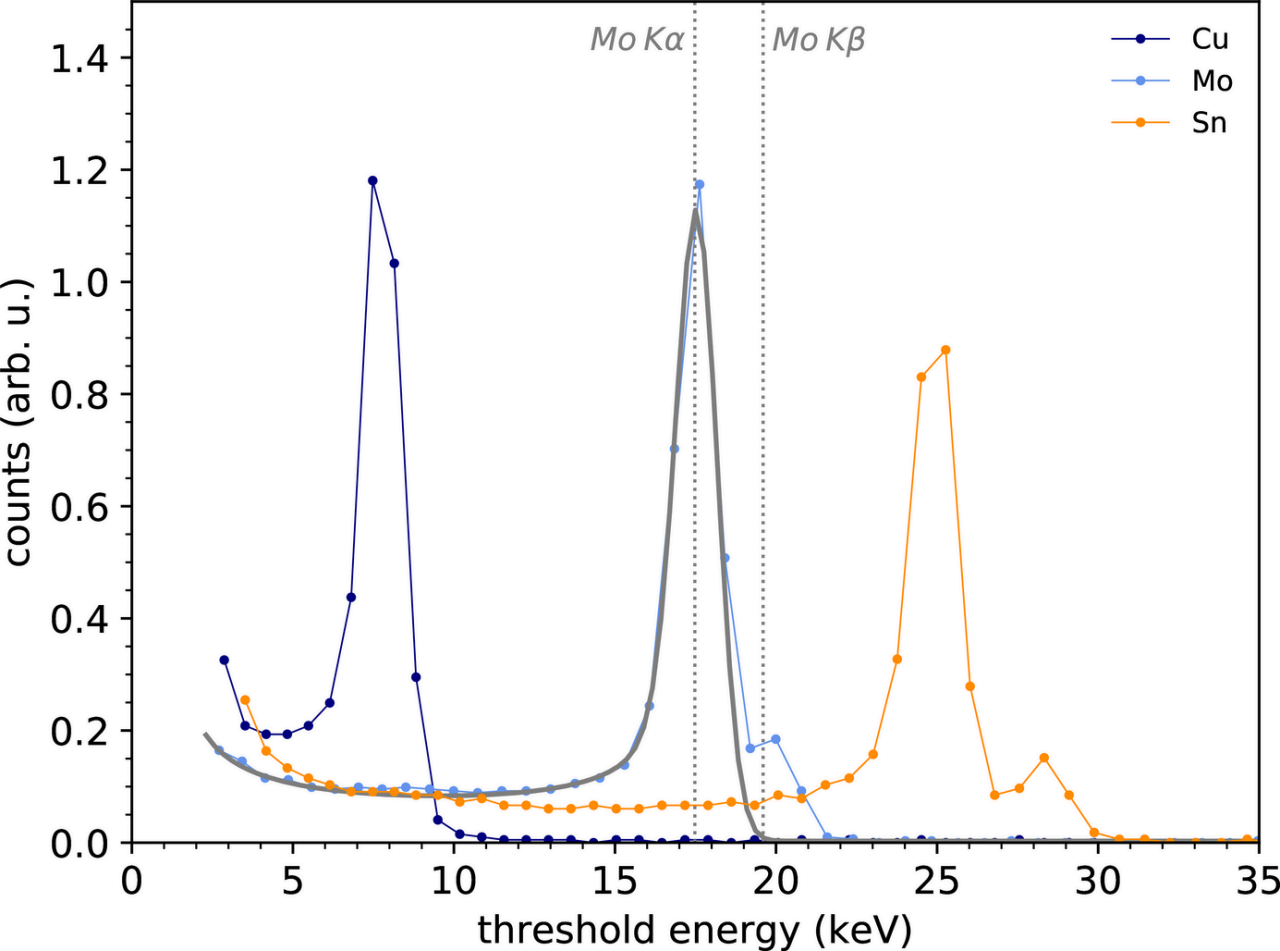
Energy spectra of Cu, Mo and Sn *K* fluorescence measured with the silicon sensor. The solid gray line represents the fitting of the Mo spectrum using the analytical pixel-response function of Zambon *et al.* (2018 ▸).

By sweeping the comparator threshold voltage it is possible to measure the integral energy spectrum of the incoming beam. By deriving it, the (differential) energy spectrum is obtained. Fig. 3 ▸ shows a series of exemplary *K*-fluorescence energy spectra measured with the silicon sensor (the median over the pixel ensemble) and obtained by illuminating Cu, Mo and Sn elemental targets with the direct beam from the X-ray tube. For a reference, the corresponding *K*α fluorescence lines lie at 8.048, 17.480 and 25.271 keV, respectively. The intensity of the spectra have been normalized to the value of the integral energy spectra at a threshold energy of half the peak energy, for visualization purposes. The energy axis has been, on the other hand, converted from comparator threshold voltage DAC units to energy thanks to the threshold energy calibration procedure highlighted in Section 3.2. The energetic separation between *K*α and *K*β increases for increasing atomic numbers, and for this reason Mo and Sn *K*β start to be visible at 19.606 and 28.485 keV, respectively. The spectrum shape contains precious information on the underlying physics of the signal formation and this can be retrieved by fitting the experimental curve with the pixel-response analytical model developed by Zambon *et al.* (2018 ▸). Taking Mo as a case study and having the care of excluding the *K*β peak from the fitting, to better fulfill the requirement of monochromaticity of the basis of the model, we achieve the result depicted as a solid gray line in Fig. 3 ▸. As fitting parameters, we obtain: (i) a charge-cloud size at the pixelated side of the sensor – responsible for the background contribution at energies lower than the *K*α and for a deterministic distortion of the peak, especially on the left-hand shoulder – of 5.3 ± 0.1 µm r.m.s.; (ii) an overall (stochastic) noise – including the contributions from the front-end electronics and the Fano noise – of 587 ± 36 eV r.m.s. Given that, in this particular case, the Fano noise^4^ amounts to 85 eV r.m.s., the contribution of the sole front-end electronics, by subtracting in quadrature, yields 580 eV r.m.s. For comparison, the overall energy resolution computed for example as the standard deviation of the Gaussian fit performed on the *K*α peak amounts to 664 eV r.m.s., slightly larger than that obtained by fitting with the more sophisticated pixel response model, as a consequence of the aforementioned spectral distortion brought by the charge-sharing phenomenon.

**Figure 4:**
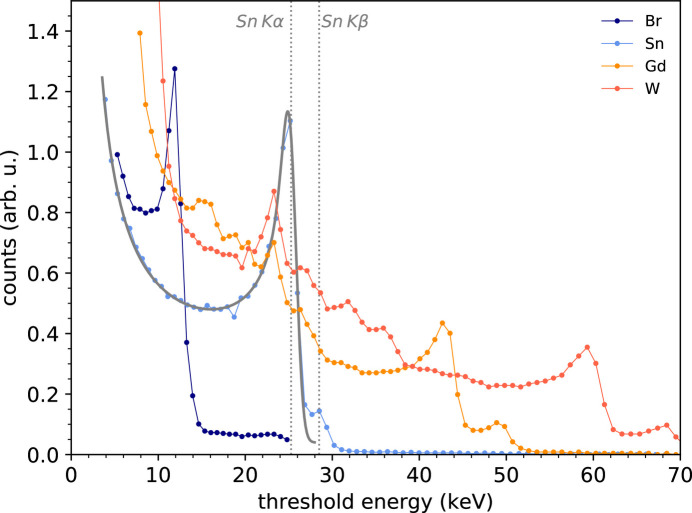
Energy spectra of Br, Sn, Gd and W *K* fluorescence measured with the CZT sensor. The solid gray line represents the fitting of the Sn spectrum using the analytical pixel-response function of Zambon *et al.* (2018 ▸).

Fig. 4 ▸ shows a series of exemplary *K*-fluorescence energy spectra measured with the CZT sensor, averaged over the pixel ensemble and obtained with Br, Sn, Gd and W elemental targets. For a reference, the corresponding *K*α fluorescence lines lie at 11.924, 25.271, 42.996 and 59.318 keV, respectively. *K*β peaks start to be visible for Sn at 28.485 keV, while are clearly distinguishable for Gd and W at 48.695 and 67.244 keV, respectively. For incoming X-ray energies above Cd and Te *K* edges – 26.711 and 31.814 keV, respectively – the recorded spectra become more complex and the appearance of several satellite peaks can be observed. They mainly consist of Cd and Te *K*α fluorescence peaks at 23.173 and 27.472 keV, respectively, and the corresponding escape peaks. Applying the pixel-response analytical model to the Sn spectrum with the exclusion of the *K*β peak, we retrieve: (i) a value of the charge-cloud size at the pixel depth of 15.1 ± 0.1 µm r.m.s., and (ii) a value of the overall noise due to the front-end electronics and the Fano noise of 640 ± 37 eV r.m.s. Given that, in this particular case, the Fano noise^5^ amounts to 100 eV r.m.s., the contribution of the sole front-end electronics, by subtracting in quadrature, yields 632 eV r.m.s. For comparison, the overall energy resolution computed with a Gaussian fit on the *K*α peak yields 1.22 keV r.m.s.

### 3.2. Threshold energy calibration

**Figure 5:**
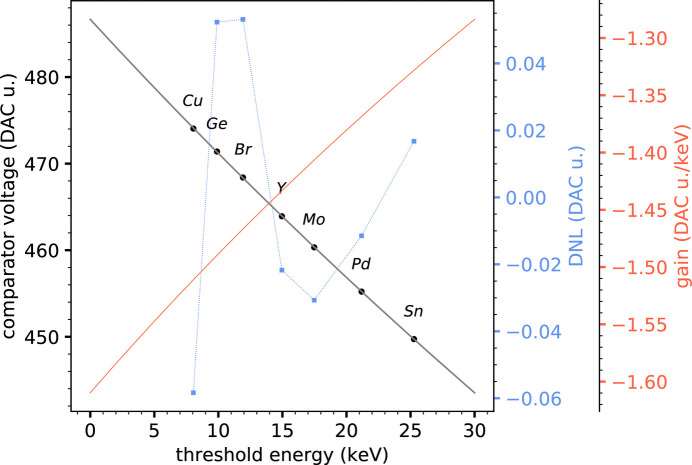
Threshold energy calibration curve for the silicon sensor. The DNL between the best-fit curve and the experimental points is also shown, together with the gain curve, which corresponds to the derivative of the calibration curve.

Figs. 5 ▸ and 6 ▸ show the threshold energy calibration curves of the silicon and CZT sensors, respectively, obtained by putting in correspondence the *K*α peak energy and the corresponding comparator threshold voltage (expressed in DAC units) of a series of elemental targets. The experimental points show a saturation effect for increasing energies, expected from the comparator stage, and it was empirically found that this non-linear behavior is well described by a hyperbolic function^6^, which was used as a fitting function. The differential non-linearity between the best-fit curves and the experimental points is also shown in the figures, as well as the gain curves, computed as the derivative of the calibration curves. The differential non-linearity (DNL) is always well below 1 DAC unit, showing a very good agreement of the experimental data with the hyperbolic function.

The knowledge of calibration curves allows us to accurately set any desired threshold within the calibrated interval and, to some extent, to safely extrapolate below and above it. For the case of the silicon sensor, we consider it safe to operate within an incoming energy range from 8 to 30 keV, while for the case of the CZT sensor within a range from 10 to 80 keV.

### 3.3. Threshold trimming

A precise threshold trimming is necessary to guarantee a uniform threshold energy value across the entire pixel array. To this purpose, we used the iterative template curve-fitting algorithm described by Zambon *et al.* (2019 ▸). To verify the goodness of this approach, we evaluated the threshold dispersion before and after the trimming procedure. The statistical distribution of the threshold values is obtained by performing a threshold scan of X-ray fluorescence radiation with and without the threshold trimming applied and by fitting the *K*α peak of the energy spectra of each individual pixel with a Gaussian function.

**Figure 7:**
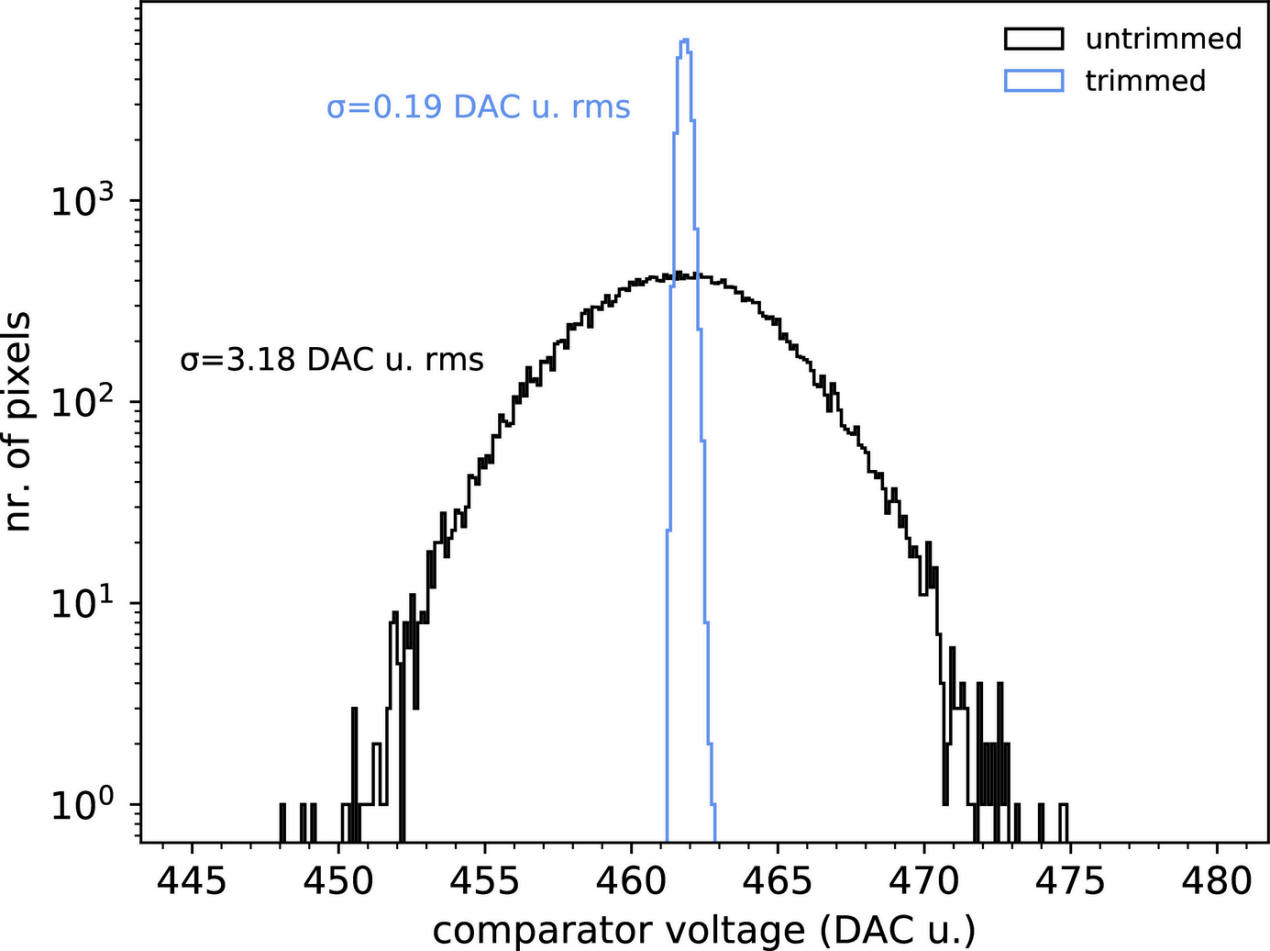
Threshold energy statistical distribution across the entire pixel matrix for the case of the silicon sensor, before and after the threshold trimming procedure, tested with Mo X-ray fluorescence. The symbol σ represents the standard deviation.

For the silicon sensor version, a Mo target was chosen. Fig. 7 ▸ shows the threshold energy histogram before and after the trimming procedure. In untrimmed conditions, the distribution has a standard deviation of 3.18 DAC u. r.m.s.; in trimmed conditions, it has a standard deviation of 0.19 DAC u. r.m.s. Translated in energy, it means 2.05 keV r.m.s. and 0.12 keV r.m.s., respectively, for an improvement of a factor 17.1.

**Figure 8:**
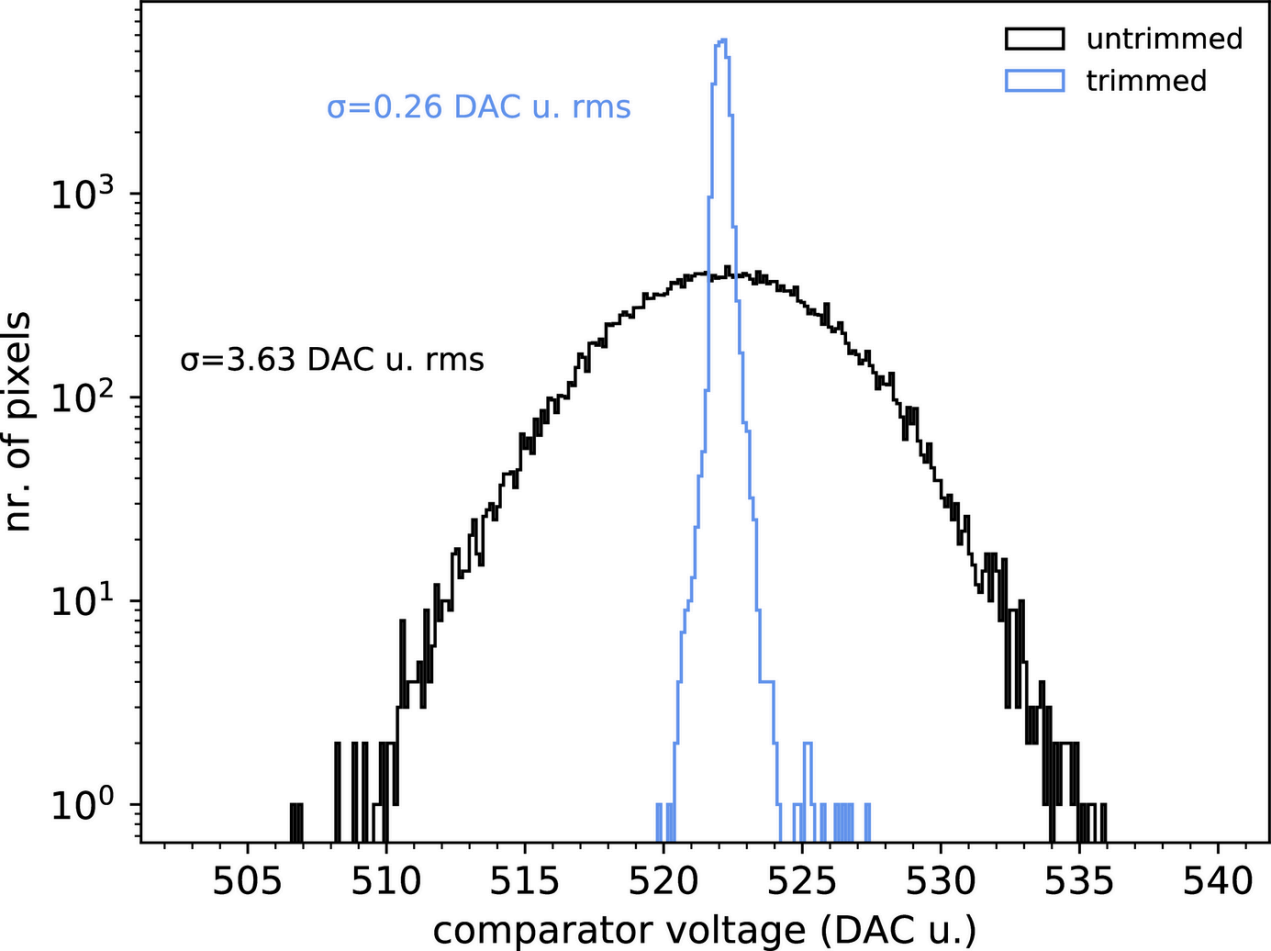
Threshold energy statistical distribution across the entire pixel matrix for the case of the CZT sensor, before and after the threshold-trimming procedure, tested with Sn X-ray fluorescence. The symbol σ represents the standard deviation.

For the CZT-sensor version, an Sn target was chosen. Fig. 8 ▸ shows the threshold energy histogram before and after the trimming procedure. In untrimmed conditions, the distribution has a standard deviation of 3.63 DAC u. r.m.s.; in trimmed conditions, it has a standard deviation of 0.26 DAC u. r.m.s. Translated in energy, it means 2.67 keV r.m.s. and 0.19 keV r.m.s., respectively, for an improvement of a factor 14.1. The slightly better performance of the silicon detector version with respect to the CZT one is due to its higher gain, which translates into a higher calibration precision.

### 3.4. Count rate performance

Count rate curves were measured by illuminating a portion of the detector area, roughly a hundred pixels, with monochromatic synchrotron light of progressively higher flux intensity, obtained by suitably decreasing the beam filtration. The incoming count rate was computed on the basis of the recorded count rate and on the assumption of linearity of the response at low flux intensities. The threshold energy was set to half the beam energy. In the following, we limit the analysis to the non-paralyzable counting mode, *i.e.* with the retrigger mechanism enabled, as it is the one intended for customary use in real-life experiments.

**Figure 9:**
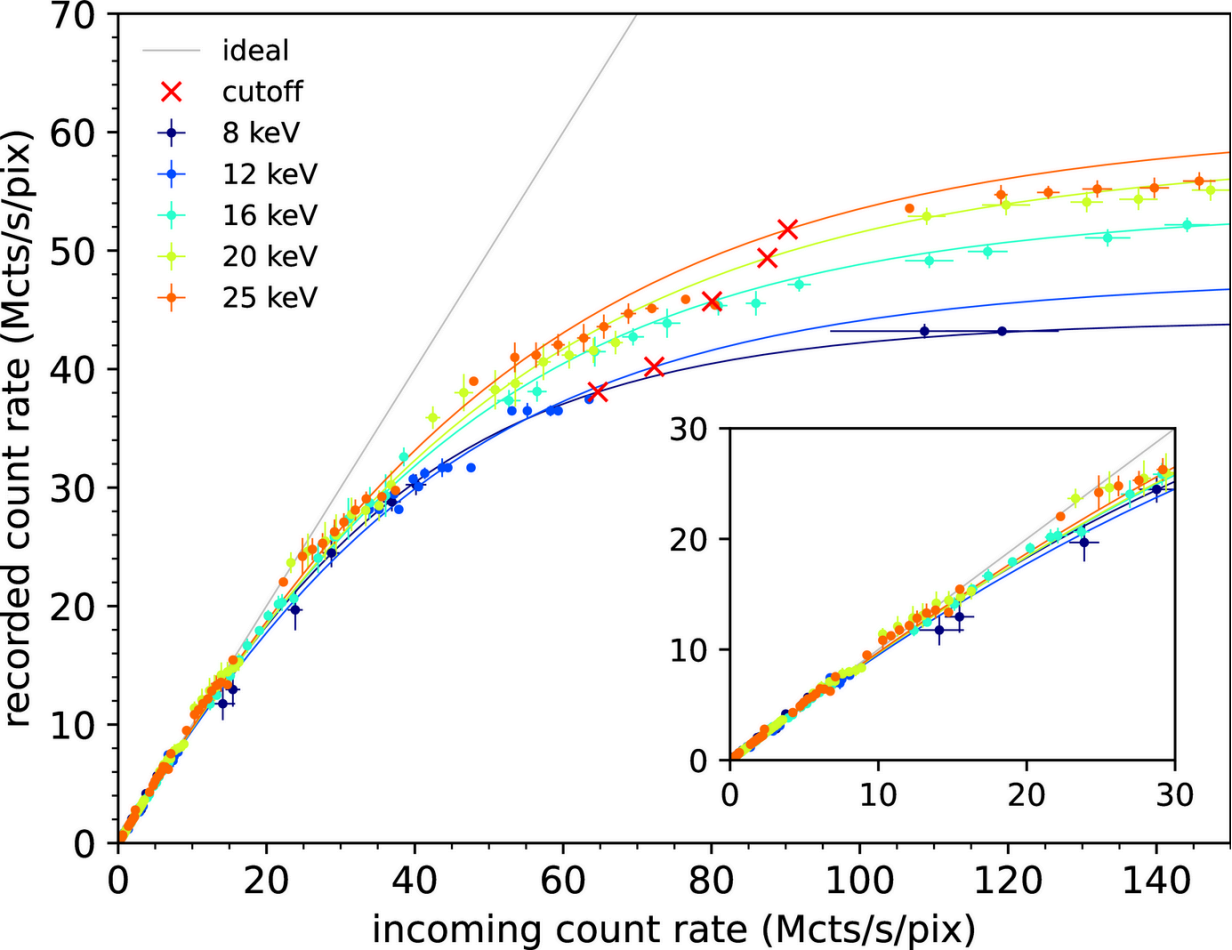
Recorded count rate as a function of the incoming count rate, using the silicon sensor and in non-paralyzable counting mode. Threshold energy is at half the beam energy. Experimental values are depicted with error bars while the fitting with the analytical model is shown as solid lines. The ideal 1:1 count rate curve and the cut-off points are also shown as reference. In the inset, a zoomed view of the linear part is shown up to 30 Mcts s^−1^ pixel^−1^.

Fig. 9 ▸ shows the count rate curves recorded with a silicon sensor as a function of the incoming count rate, for several values of X-ray energies. Experimental values are displayed as error bars. The peculiar non-paralyzable counting mode enabled by the retrigger mechanism can be described analytically with the function derived by Zambon (2021 ▸), 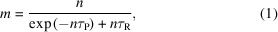
where *n* is the incoming count rate, *m* is the recorded count rate and τ_P_ is a quantity related to the temporal width of the analog pulse. In the context of non-ideal (non-rectangular) signal pulses, it can be thought of as an effective value for the temporal duration of the signal. In any case, for values of incoming rate tending to infinity, the recorded count rate tends to 1/τ_R_. Incidentally, and for reference, it is worth mentioning that not only the average recorded count rate can be analytically modeled but also its temporal and spatial variance (Zambon, 2022 ▸). Equation (1) was used as a fitting function, yielding the curves depicted as solid lines. The values of the fitting parameters τ_R_ and τ_P_ are reported in Table 2 ▸, along with two further figures of merit. The first is the value of the incoming count rate leading to a 10% recorded count rate loss, which can be considered as a measure of the response linearity. The other is the ‘cut-off’, which can be considered as an upper limit for the count rate correction, expressed in incoming count rate units. Beyond this point, indeed, the accuracy of the rectification is limited by unavoidable pixel-to-pixel and chip-to-chip variations and, based on our experience, it was defined as the point where the derivative of the count rate curves assumes a (arbitrary) value of 0.2. Values of the 10% recorded count rate loss range from a minimum of 16.36 Mcts s^−1^ pixel^−1^ at 12 keV to a maximum of 24.32 Mcts s^−1^ pixel^−1^ at 25 keV, while values of the cut-off range from a minimum of 64.67 Mcts s^−1^ pixel^−1^ at 8 keV up to a maximum of 90.28 Mcts s^−1^ pixel^−1^ at 25 keV. The reason for the general increasing behavior as a function of the incoming energy, with exception made for the 10% count loss at 12 keV that is most probably due to measurement inaccuracies, arises from the slight narrowing of the analog-signal shape as a consequence of the front-end electronics non-linearity, as confirmed by schematic-level simulations.

**Figure 10:**
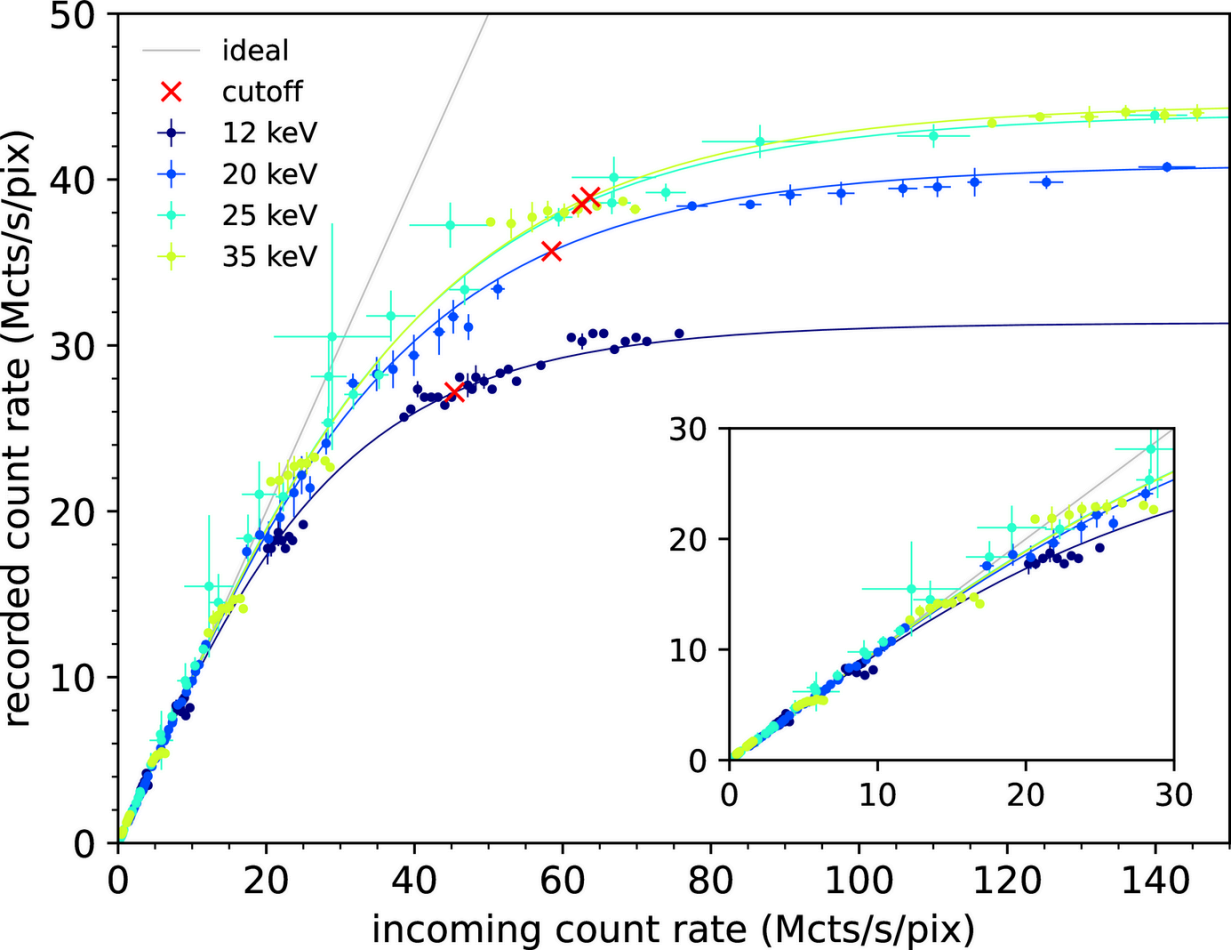
Recorded count rate as a function of the incoming count rate, using the CZT sensor and in non-paralyzable counting mode. Threshold energy is at half the beam energy. Experimental values are depicted with error bars while the fitting with the analytical model is shown as solid lines. The ideal 1:1 count rate curve and the cut-off points are also shown as reference. In the inset, a zoomed view of the linear part is shown up to 30 Mcts s^−1^ pixel^−1^.

Fig. 10 ▸ shows the count rate curves recorded with a CZT sensor as a function of the incoming count rate, for several values of X-ray energies. Experimental values are displayed as error bars, while the fitting with the analytical model is shown as solid lines. Table 3 ▸ summarizes the fitting parameters τ_R_ and τ_P_, the incoming count rate leading to 10% recorded count rate loss, and the cut-off values. Values of the 10% recorded count rate loss range from a minimum of 15.21 Mcts s^−1^ pixel^−1^ at 12 keV to a maximum of 23.52 Mcts s^−1^ pixel^−1^ at 35 keV, while values of the cut-off range from a minimum of 45.41 Mcts s^−1^ pixel^−1^ at 12 keV up to a maximum of 63.71 Mcts s^−1^ pixel^−1^ at 25 keV. The same reasoning on the monotonicity as a function of the beam energy as for the case of the silicon sensor applies.

## 4. Conclusions

In this work, we have introduced SELUN, a novel X-ray counting detector based on hybrid pixel technology, whose design, driven towards high count rate and high frame rate capabilities, makes it particularly suitable for coherent X-ray imaging applications.

The device features a matrix of 192 × 192 pixel elements, each with a physical size of 100 µm × 100 µm. The bipolar front-end electronics are compatible with both the hole-collection mode required by standard silicon sensors and the electron-collection mode required by high-*Z* sensors for best matching with the intended X-ray energy range. While the instant retrigger technology allows the system to work in a peculiar non-paralyzable counting mode, sensibly extending the response linear range towards higher values, two independent on-chip data compression mechanisms, namely floating-point counter encoding and 2 × 2 pixel digital binning, allow for frame rates as high as 120 kfps.

Our characterization campaign investigated the energy resolution of the energy threshold of a detector system equipped with a silicon sensor and one with a CZT sensor. The system with the silicon sensor showed an energy resolution, measured at Mo *K*α fluorescence peak, of 664 eV r.m.s., arising from the contribution of a pure electronic noise of 587 eV r.m.s. and charge cloud size at the pixel depth of 5.3 µm. The threshold energy dispersion was, after the trimming procedure, 120 eV r.m.s. Count rate measurements showed that a 10% recorded count rate loss occurs for incoming count rates in the range 16–24 Mcts s^−1^ pixel^−1^, while cut-off values occur for incoming count rates in the range 64–90 Mcts s^−1^ pixel^−1^, depending on the X-ray energy. The system with the CZT sensor showed, on the other hand, an energy resolution, measured at Sn *K*α fluorescence peak, of 1.22 keV r.m.s., with a contribution from the pure electronic noise of 640 eV r.m.s. and charge cloud size at the pixel depth of 15.1 µm. The threshold energy dispersion was, after the trimming procedure, 190 eV r.m.s. The 10% recorded count rate loss happens for incoming count rates in the range 15–23 Mcts s^−1^ pixel^−1^, while cut-off values occur for incoming count rates in the range 45–63 Mcts s^−1^ pixel^−1^, depending on the X-ray energy.

Overall, we have presented this new hybrid pixel detector SELUN and evaluated its principal performance parameters. Its combination of high count rate capability and frame rates up to 120 kHz, using on-chip data compression, makes it well suited for applications in coherent diffractive imaging and XPCS experiments at modern synchrotron sources.

## Figures and Tables

**Figure 6:**
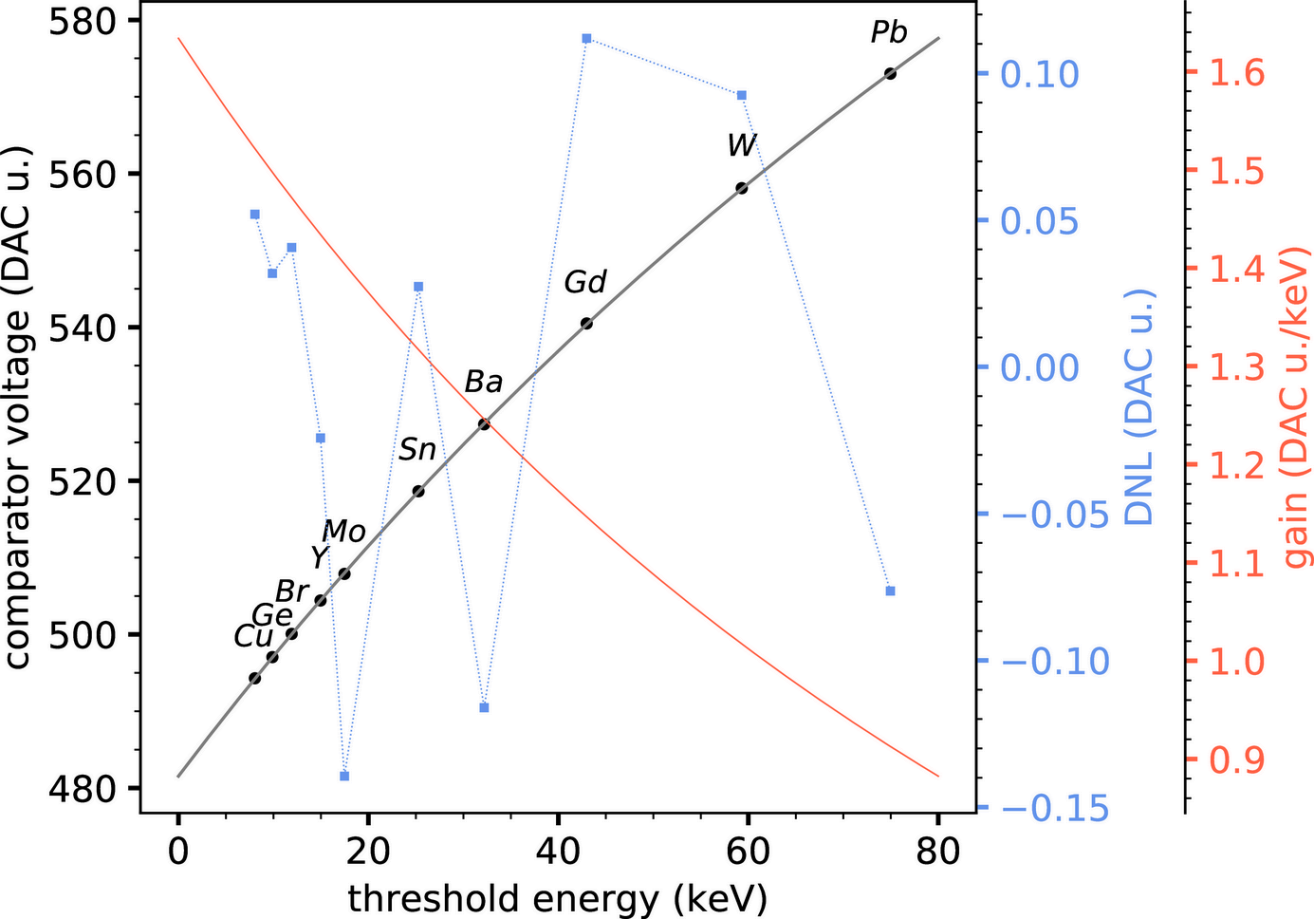
Threshold energy calibration curve for the CZT sensor. The DNL between the best-fit curve and the experimental points is also shown, together with the gain curve, which corresponds to the derivative of the calibration curve.

**Table 1:** Key technical parameters of the ASIC

Power supply voltage	1.2 V
Pixel size	100 µm × 100 µm
Pixel array	192 × 192 pixels
Active area	19.2 mm × 19.2 mm
Die size (including pads)	20.5 mm × 20.5 mm
Operating mode	Photon counting
Input polarity	Positive (Si)/negative (high *Z*)
Number of thresholds	1
Instant retrigger	Yes
Continuous readout	Yes
Readout modes	Full frame/2 × 2 pixel digital binning
Counter encoding	12-bit integer/8-bit floating point
Operating temperature	Room temperature
Static power dissipation	1.05 W
Dynamic power dissipation	<4 W

**Table 2:** Count rate parameters – Si sensor

Beam energy (keV)	Threshold energy (keV)	10% loss (Mcts s^−1^ pixel^−1^)	τ_R_ (ns)	τ_P_ (ns)	Cut-off (Mcts s^−1^ pixel^−1^)
8	4	19.96	22.61 ± 0.07	22.31 ± 1.24	64.67
12	6	16.36	20.89 ± 0.01	17.21 ± 0.02	72.28
16	8	21.02	18.60 ± 0.10	16.71 ± 0.52	80.07
20	10	21.50	17.12 ± 0.06	14.80 ± 0.51	87.54
25	12.5	24.32	16.66 ± 0.01	13.54 ± 0.01	90.28

**Table 3:** Count rate parameters – CZT sensor

Beam energy (keV)	Threshold energy (keV)	10% loss (Mcts s^−1^ pixel^−1^)	τ_R_ (ns)	τ_P_ (ns)	Cut-off (Mcts s^−1^ pixel^−1^)
12	6	15.21	31.87 ± 0.01	33.00 ± 0.01	45.41
20	10	21.38	24.44 ± 0.07	26.67 ± 0.52	58.5
25	12.5	23.81	22.71 ± 0.14	25.41 ± 0.97	62.58
35	17.5	23.52	22.41 ± 0.01	24.62 ± 0.01	63.71

## Footnotes

1 The signal is digitized through thresholding and counting directly in the pixel, and its readout is purely a digital operation.

2 Provided that the threshold energy is sufficiently higher than the noise floor.

3 The switching between counters has a negligible time overhead.

4 Assuming a Fano factor of 0.115 and an electron–hole pair creation energy of 3.61 eV.

5 Assuming a Fano factor of 0.089 and an electron–hole pair creation energy of 4.5 eV.

6 The used function was in the form , where *a*, *b*, *c* and *d* are fitting parameters.
